# Negative- versus positive-pressure ventilation in intubated patients with acute respiratory distress syndrome

**DOI:** 10.1186/cc11216

**Published:** 2012-03-02

**Authors:** Konstantinos Raymondos, Ulrich Molitoris, Marcus Capewell, Björn Sander, Thorben Dieck, Jörg Ahrens, Christian Weilbach, Wolfgang Knitsch, Antonio Corrado

**Affiliations:** 1 Anaesthesiology and Intensive Care Medicine, Medical School Hanover, Carl-Neuberg-Strasse 1, D-30625 Hanover, Germany; 2 Anaesthesiology, St-Josefs-Hospital, Krankenhausstraße 13, D-49661 Cloppenburg, Germany; 3 General, Visceral and Transplantation Surgery, Medical School Hanover, Hanover, Carl-Neuberg-Strasse 1, D-30625 Hanover, Germany; 4 Unita' di Terapia Intensiva Pneumologica e, Fisiopatologia Toracica, DAI, Specialità medico-Chirurgiche, Azienda Ospedaliero-Universitaria Careggi, Padiglione San Luca, Via di San Luca 1, I-50136 Florence, Italy

**Keywords:** iron lung, tank respirator, external negative-pressure ventilation, acute lung injury

## Abstract

**Introduction:**

Recent experimental data suggest that continuous external negative-pressure ventilation (CENPV) results in better oxygenation and less lung injury than continuous positive-pressure ventilation (CPPV). The effects of CENPV on patients with acute respiratory distress syndrome (ARDS) remain unknown.

**Methods:**

We compared 2 h CENPV in a tankrespirator ("iron lung") with 2 h CPPV. The six intubated patients developed ARDS after pulmonary thrombectomy (*n *= 1), aspiration (*n *= 3), sepsis (*n *= 1) or both (*n *= 1). We used a tidal volume of 6 ml/kg predicted body weight and matched lung volumes at end expiration. Haemodynamics were assessed using the pulse contour cardiac output (PiCCO) system, and pressure measurements were referenced to atmospheric pressure.

**Results:**

CENPV resulted in better oxygenation compared to CPPV (median ratio of arterial oxygen pressure to fraction of inspired oxygen of 345 mmHg (minimum-maximum 183 to 438 mmHg) vs 256 mmHg (minimum-maximum 123 to 419 mmHg) (*P *< 0.05). Tank pressures were -32.5 cmH_2_O (minimum-maximum -30 to -43) at end inspiration and -15 cmH_2_O (minimum-maximum -15 to -19 cmH_2_O) at end expiration. NO Inspiratory transpulmonary pressures decreased (*P *= 0.04) and airway pressures were considerably lower at inspiration (-1.5 cmH_2_O (minimum-maximum -3 to 0 cmH_2_O) vs 34.5 cmH_2_O (minimum-maximum 30 to 47 cmH_2_O), *P *= 0.03) and expiration (4.5 cmH_2_O (minimum-maximum 2 to 5) vs 16 cmH_2_O (minimum-maximum 16 to 23), *P *=0.03). During CENPV, intraabdominal pressures decreased from 20.5 mmHg (12 to 30 mmHg) to 1 mmHg (minimum-maximum -7 to 5 mmHg) (*P *= 0.03). Arterial pressures decreased by approximately 10 mmHg and central venous pressures by 18 mmHg. Intrathoracic blood volume indices and cardiac indices increased at the initiation of CENPV by 15% and 20% (*P *< 0.05), respectively. Heart rate and extravascular lung water indices remained unchanged.

**Conclusions:**

CENPV with a tank respirator improved gas exchange in patients with ARDS at lower transpulmonary, airway and intraabdominal pressures and, at least initially improving haemodynamics. Our observations encourage the consideration of further studies on the physiological effects and the clinical effectiveness of CENPV in patients with ARDS.

## Introduction

Acute respiratory distress syndrome (ARDS) is usually treated with invasive continuous positive-pressure ventilation (CPPV) [1], which can aggravate both lung injury and multisystem organ failure [2]. Studies of mechanical ventilation in patients with ARDS have focused on low tidal volume and high positive end-expiratory pressure (PEEP) [2-5]. Less injurious low tidal volume can lead to impaired oxygenation [4], and even very high PEEP can be insufficient to maintain lung volume in patients with severe ARDS [3]. Other approaches using mechanical ventilation have not been shown to further improve outcome, and mortality in patients with ARDS still reaches 50% [1].

External negative-pressure ventilation with tank respirators is very successful in treating patients with chronic obstructive pulmonary disease [6], but there are no data regarding patients with hypoxaemic acute respiratory failure. Recent experimental data suggest that continuous external negative-pressure ventilation (CENPV) may distend lungs in a fundamentally different manner from CPPV and may result in better oxygenation and less lung injury at lower transpulmonary pressures [7]. Extrapolation to patients is difficult, and to date only continuous external negative-pressure (CENP) has been applied in three ARDS patients who breathed spontaneously in Emerson tank respirators [8-10]. Furthermore, cuirass [11] or poncho wrap systems [12,13] have been used for CENP during intermittent positive-pressure ventilation (IPPV), which resulted in improved cardiac output [11-13]. However, both cuirass and poncho wrap systems decrease chest wall compliance when they are affixed to the body [11-13], and effective ventilation in patients with ARDS has not been reported with either these systems or with tank respirators.

We speculated that, similarly to recent experimental data, CENPV with a tank respirator would also result in better oxygenation in intubated patients with ARDS, even when low tidal volumes were used. Therefore, we performed a physiologic study to compare CENPV with CPPV using matched lung volumes at end expiration and matched low tidal volumes. Favourable physiologic effects may help to promote CENPV as an applicable and even noninvasive ventilatory mode for patients with ARDS.

## Materials and methods

The study was approved by the ethical committee of our institution, and informed consent was obtained from the patients' next-of-kin. We studied six intubated patients between January 2001 and January 2002. Technical personnel and approaches did not change during the study period. Within 12 hours before study entry, all patients met ARDS criteria [14]. Their clinical characteristics, Simplified Acute Physiology Score II scores [15] and respiratory settings at that time are listed in Table 1. The patients were placed under sedation analgesia and did not breathe spontaneously. We performed a recruitment manoeuvre as described below to standardise the history of lung volume [16], and, to achieve more comparable conditions, we adjusted PEEP to 16 cmH_2_O in patients 1 through 5 (and to 23 cmH_2_O in patient 6). The patients were ventilated with a lung-protective strategy using a tidal volume of 6 ml/kg predicted body weight (as calculated in [4]).

**Table 1:** Clinical characteristics and respiratory variables of the patients within 12 hours before study entry^a^

	Cause of lung injury		Demographics		Respiratory variables							
			
Patient	Disorders predisposing to ARDS	Underlying disease	SAPS II	Body mass index	PaO_2_/FiO_2 _ratio	Plateau pressure (cmH_2_O)	PEEP (cmH_2_O)	FiO_2_	PaCO_2 _(mmHg)	pH	Days on ventilator	Outcomes
1	Aspiration	Brain injury	34	29.3	152	26	9	0.4	41	7.39	16	Deceased
2	Severe pulmonary thromboembolism and thrombectomy	Parkinson's disease	33	25.7	190	32	10	0.4	46	7.40	3	Survived
3	Sepsis, liver failure after valproate administration	Endometritis, epilepsy	35	31.1	153	30	14	0.5	45	7.43	5	Survived
4	Aspiration	Subarachnoid haemorrhage	33	27.6	190	28	9	0.5	44	7.48	3	Deceased
5	Aspiration	Gastric ulcer perforation	34	29.9	153	36	13	0.6	49	7.32	2	Survived
6	Sepsis, aspiration	Colon diverticulitis	42	30.4	118	46	22	0.8	65	7.30	47	Deceased
Mean ± SD			35 ± 3	29 ± 2	159 ± 27	33 ± 7	13 ± 5	0.5 ± 02	48 ± 9	7.39 ± 0.07	18 ± 11	

^a^Patient 2 developed ARDS after embolectomy and three days of mechanical ventilation at pulmonary artery wedge pressures below 18 mmHg. Patient 6 developed ARDS already at admission due to a combination of sepsis after bowel suture insufficiency and aspiration and was treated with lung-protective ventilation already for 47 days at study entry. ARDS, acute respiratory distress syndrome; FiO_2_, fraction of inspired oxygen pressure; PaCO_2_, arterial carbon dioxide pressure; PaO_2_/FiO_2_, arterial oxygen-to-fraction of inspired oxygen pressure ratio; PEEP, positive end-expiratory pressure; SAPS II, Simplified Acute Physiology Score II [15].

We compared 2-hour CENPV using a tank respirator with 2-hour CPPV using biphasic positive airway pressure/airway pressure release ventilation with an Evita 1 ventilator (Dräger, Lübeck, Germany). We randomised the sequence of the ventilatory mode to balance the effects of the previous ventilation period. The six patients were randomized to receive 2 hours of CENPV first and then 2 hours of CPPV (*n *= 3 patients) or 2 hours of CPPV first and then 2 hours of CENPV (*n *= 3 patients) in an unchanged supine position. Between both ventilatory modes, we returned the ventilation, ventilation is the correct word to baseline, and the whole experiment lasted 6 to 7 hours.

We matched tidal volume, respiratory frequency and the lung volume at end expiration that represented the difference between functional residual capacity (FRC) with and without end-expiratory pressure. The ratio of inspiration to expiration was 1:1. After a recruitment manoeuvre of six deep breaths with an inspiratory pressure of 60 cmH_2_O, the lung volume at end expiration was measured by a sudden release of the relevant positive or negative pressure at end expiration with a spirometer (Volumeter 3000; Dräger). This step was repeated two more times, and the average value of the three measurements was calculated. Next, lung volumes at end expiration of the second ventilation mode were matched to those measured with the first one. To find out the corresponding pressure at end expiration, we first measured the lung volume at a similar positive or negative pressure. We then increased or decreased pressure according to the achieved volumes and repeated the measurement manoeuvre until the difference between lung volumes at end expiration was smaller than 50 ml. We then performed three measurements again as described above.

To achieve comparable conditions, we recruited the lungs again immediately before CPPV or CENPV with 25 deep breaths applied during 1 minute using inspiratory peak pressures of 60 cmH_2_O. We performed this manoeuvre to standardise the history of lung volume [16] to improve the comparability between both ventilatory modes. We used a time interval of 1 minute to allow us to take haemodynamic measurements during the recruitment manoeuvre using the PiCCO system (Pulsion Medical Systems, Munich, Germany).

**Figure 1:**
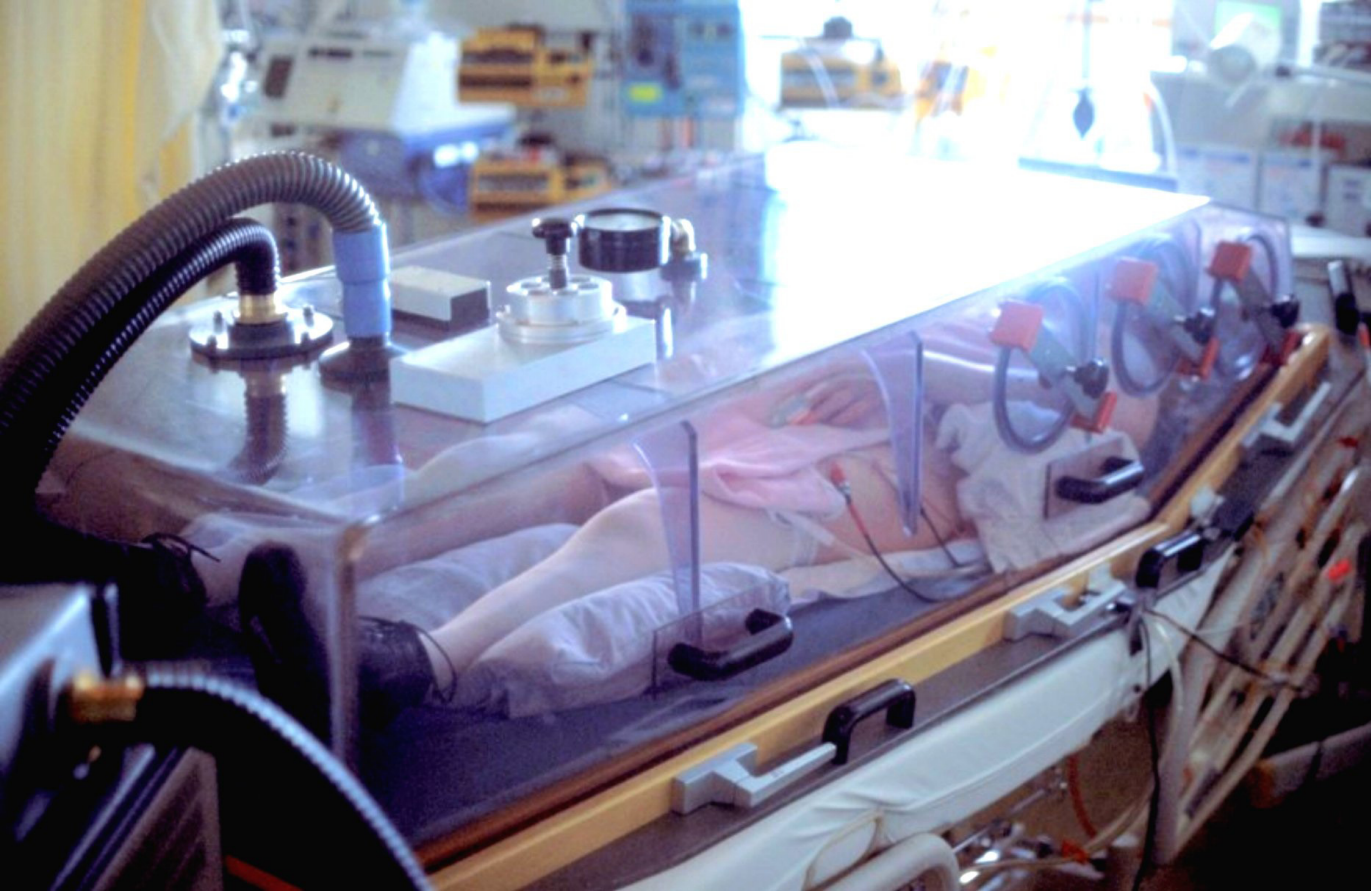
**The transparent plastic tank respirator during ventilation of the second patient (Table 1)**. The tank covered the whole patient, including the head. This setting improves the practicability of continuous external negative-pressure ventilation in an intubated patient in whom flow is delivered from the conventional mechanical ventilator through the endotracheal tube. Apertures in the bottom, below the wooden frame, were used to lead out all connections to the patient, and trimmed-to-fit sponge rubbers were used to seal these apertures. (The shoes were put on this patient to prevent contractions.).

Thanks to an initiative of Prof Ina Pichlmayr the tank respirator and the pump aggregate to generate negative pressures had been manufactured at our institution during the early 1980s [17]. The transparent plastic tank has been used in various clinical settings in patients without endotracheal tubes [18] with the head placed outside the tank as commonly practiced [6-10]. As we were studying intubated patients, it was not necessary to place the head outside the tank (Figure 1). Covering the whole body including the head avoided several problems such as air leakage at the neck and improved the practicability of using the tank respirator. Nursing care was very limited, and therefore the whole tank was removed when complex nursing care was necessary. Similar to classic tank respirators [6-8], apertures at both sides allowed access to the patients, which enabled the use of nursing procedures such as endotracheal tube suctioning. In case of an emergency such as cardiac arrest, the plastic tank can be removed within a few seconds.

During CENPV, the inspiratory changes of airway flow caused by the tank respirator triggered the conventional mechanical ventilator that delivered flow at a peak inspiratory pressure of 5 cmH_2_O in the pressure support mode of the Evita 1 ventilator. The conventional ventilator was set to 0 cmH_2_O at end expiration. The ventilatory circuits, arterial and central venous lines, gastric tube, urine catheter, electrocardiographic leads, pulse oximeters and other connections to the patient were led out of the tank via apertures from its bottom.

Airway pressures were measured via the side hole of a modified Swan-Ganz catheter that was introduced via the endotracheal tube and placed into the trachea 1 cm distal to the tip of the tube. Oesophageal pressures were measured with a conventional balloon catheter system (CP-100 Pulmonary Monitor; BiCore Monitoring Systems, Irvine, CA, USA). The oesophageal balloon catheter was passed to a depth of 60 cm, and placement of the balloon in the stomach was confirmed by a transient increase in pressure during gentle compression of the abdomen. We then withdrew the catheter until oesophageal placement was confirmed by the presence of cardiac artefacts and pressure changes during tidal ventilation [16]. Intraabdominal pressure was obtained by measuring the pressure in the bladder via the urine catheter after filling the empty bladder with 50 ml of saline using the midaxillary level as the reference line [19]. All pressure measurements were referenced to atmospheric pressure outside the tank [20].

Cardiac index, intrathoracic blood volume index, extravascular lung water index and stroke volume variation were assessed by thermal dilution using the PiCCO system with an arterial catheter inserted into a femoral artery. The Wilcoxon signed-rank test was used to compare values between CENPV and CPPV, and *P*-values less than 0.05 were considered significant.

## Results

### Gas exchange

**Figure 2:**
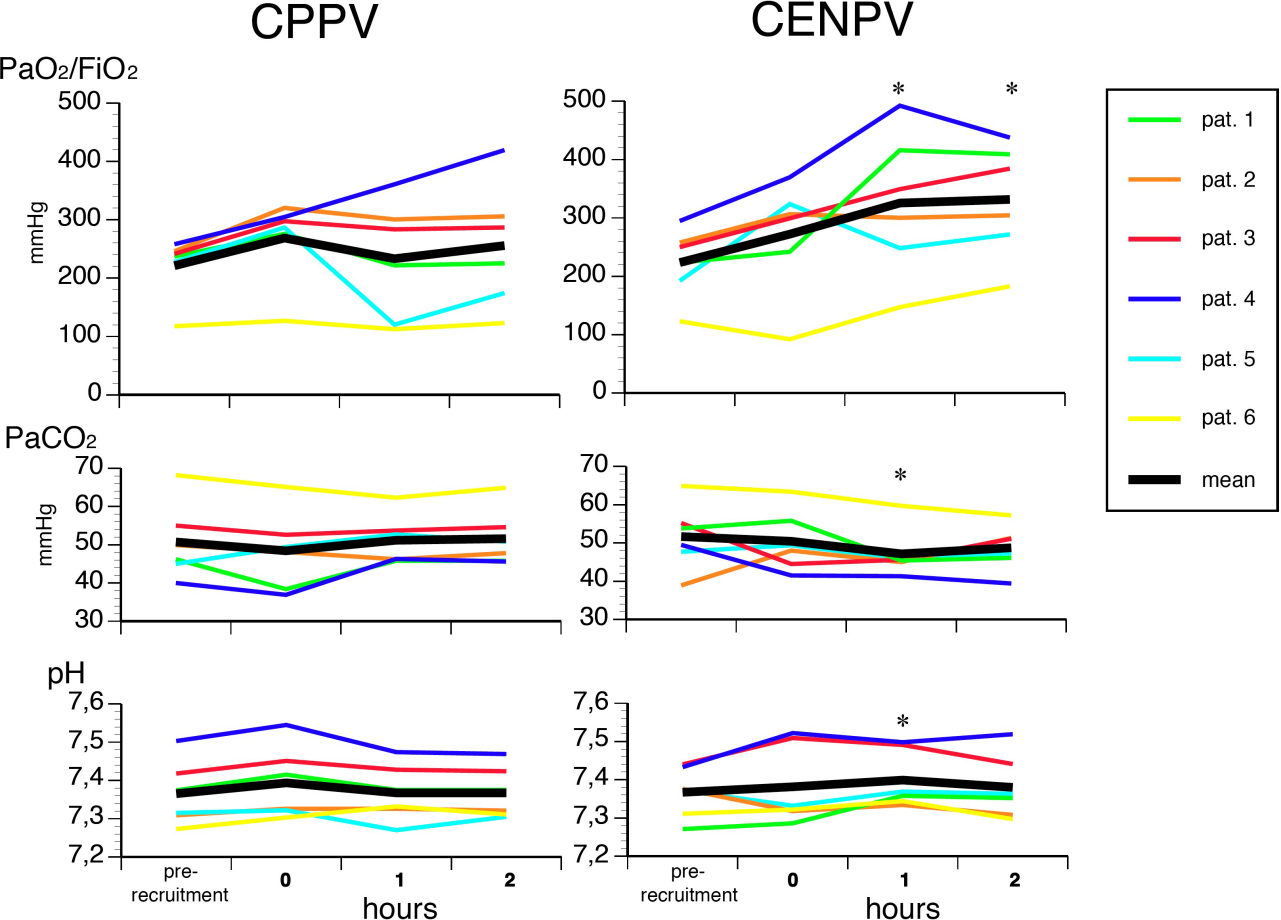
**The course of arterial oxygen-to-fraction of inspired oxygen pressure ratio (PaO_2_/FiO_2_), arterial carbon dioxide partial pressure (PaCO_2_) and pH immediately before lung recruitment and during continuous positive-pressure ventilation (CPPV) and continuous external negative-pressure ventilation (CENPV)**. Measurements were taken at time 0 (5 minutes after the recruitment manoeuvre) immediately after starting the 2-hour ventilatory period of CPPV or CENPV. *P < 0.05 compared to corresponding values at 1 or 2 hours during CPPV.

At the beginning of CENPV and CPPV, gas exchange was similar (Figure 2). During CENPV, oxygenation improved impressively compared to the corresponding values during CPPV. The mean arterial-to-inspired oxygen pressure ratio (PaO_2_/FiO_2 _ratio) increased by 92 mmHg (40%) after 1 hour and by 76 mmHg (30%) after 2 hours. However, the individual responses varied considerably between patients during both CPPV and CENPV (Figure 2). Furthermore, arterial carbon dioxide pressure (PaCO_2_) decreased and pH increased during CENPV, but statistical significance was reached only at 1 hour (Figure 2).

### Respiratory mechanics

**Figure 3:**
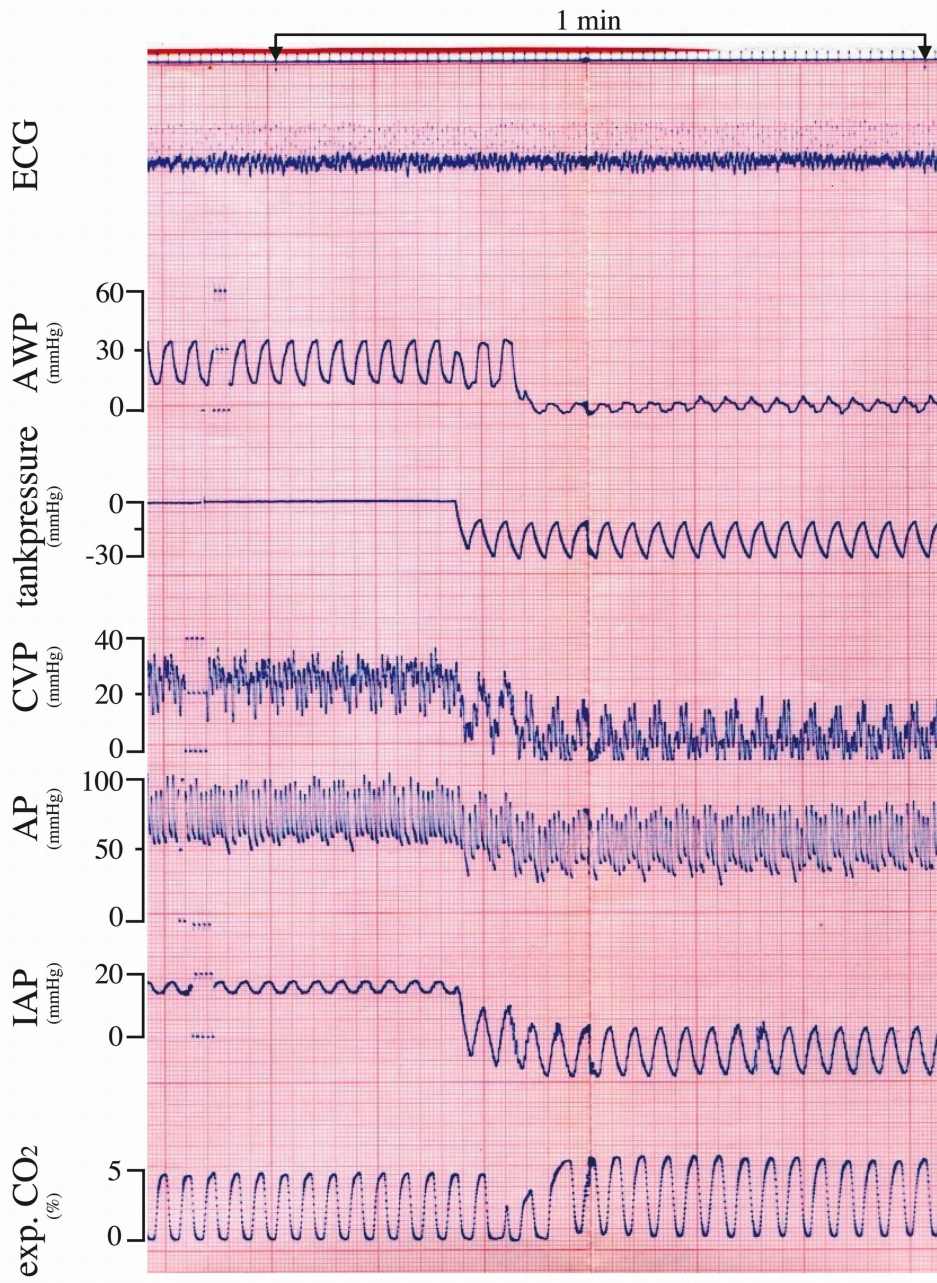
**Original polygraph recordings during a change from continuous positive-pressure ventilation (CPPV) to continuous negative-pressure ventilation (CENPV) in patient 6**. The pressure-time profiles of endotracheal pressure (AWP) during CPPV and tank pressure were similar during inspiration and expiration. During CENPV, endotracheal airway pressure increased during inspiration and decreased after a short initial peak. This patient had high intraabdominal pressure despite an open abdomen that decreased impressively during CENPV. (To convert pressure values from millimetres of mercury to centimetres of water, multiply by 1.33.) ECG, electrocardiogram; AWP, airway pressure (measured in the trachea); CVP, central venous pressure; AP, arterial pressure; IAP, intraabdominal pressure, exp. CO_2_, expired carbon dioxide.

Individual data of each patient regarding lung volumes and pressures are shown in Tables 2 and 3. Lung volumes were well-matched and did not differ between CPPV and CENPV (Table 2). During CENPV, intraabdominal pressures decreased by 15 to 26 mmHg (Table 2 Figure 3). Endotracheal airway pressures decreased by at least 30 cmH_2_O at inspiration and by at least 11 cmH_2_O at expiration (Figure 3), and inspiratory transpulmonary pressures (airway pressure minus oesophageal pressure) were also significantly lower during CENPV (Table 3). In contrast, transrespiratory system pressures (airway pressure minus tank pressure) during CENPV were similar at inspiration and 1 to 4 cmH_2_O higher at expiration (Table 3) as a result of a short peak of endotracheal airway pressure at the beginning of expiration (Figure 3).

**Table 2:** Lung volume and intraabdominal pressure during continuous positive-pressure ventilation and continuous external negative-pressure ventilation^a^

	Tidal volume (ml)		Minute volume (L/minute)			Lung volume at end expiration (ml)		Intraabdominal pressure (mmHg)	
			
Patient	CPPV	CENPV	CPPV	CENPV	Respiratory rate (breaths/minute)	CPPV	CENPV	CPPV	CENPV
1	583	578	10.5	10.4	18	612	623	22	4
2	411	417	7.4	7.5	18	623	607	28	5
3	550	559	12.1	12.3	22	937	927	12	-6
4	494	506	8.4	8.6	17	710	722	30	5
5	437	426	8.3	8.1	19	545	530	13	-2
6	560	568	14	14.2	25	667	650	19	-7
Mean ± SD	506 ± 70	509 ± 72	10.1 ± 2.6	10.2 ± 2.6	19.8 ± 3.1	682 ± 137	677 ± 138	21 ± 8	0 ± 6*

^a^CENPV, continuous external negative-pressure ventilation; CPPV, continuous positive-pressure ventilation. Patient 6 had an open abdomen. *P = 0.03 compared to CPPV.

**Table 3:** Ventilatory pressures during continuous positive-pressure ventilation and continuous external negative-pressure ventilation at inspiration and expiration^a^

	Airway pressure (cmH_2_O)		Tank pressure (cmH_2_O)	Transrespiratory pressure (cmH_2_O)	Oesophageal pressure (cmH_2_O)		Transpulmonary pressure (cmH_2_O)	
	
Patient	CPPV	CENPV	CENPV	CENPV	CPPV	CENPV	CPPV	CENPV
1								
Inspiration	33	-3 (27)	-30	27	20	-9 (21)	13	6
Expiration	16	5 (20)	-15	20	15	4 (19)	1	1
2								
Inspiration	36	-2 (31)	-33	31	22	-9 (24)	14	7
Expiration	16	5 (20)	-15	20	15	5 (20)	1	0
3								
Inspiration	30	0 (31)	-31	31	19	-8 (23)	11	8
Expiration	16	2 (17)	-15	17	15	2 (17)	1	0
4								
Inspiration	33	-1 (31)	-32	31	25	-9 (23)	8	8
Expiration	16	4 (19)	-15	19	16	4 (19)	0	0
5								
Inspiration	39	-2 (38)	-40	38	23	-10 (30)	16	8
Expiration	16	4 (19)	-15	19	16	3 (18)	0	1
6								
Inspiration	47	0 (43)	-43	43	32	-10 (33)	15	10
Expiration	23	5 (24)	-19	24	22	5 (24)	1	0
Mean ± SD								
Inspiration	36 ± 6	-1.3 ± 1.2* (33.5 ± 5.9)	-35 ± 5	34 ± 6	24 ± 5	-9 ± 0.8* (25.7 ± 4.7)	13 ± 3	8 ± 1.3^†^
Expiration	17 ± 3	4.2 ± 1.2* (19.5 ± 2.3)	-16 ± 1.6	20 ± 2*	17 ± 3	4 ± 1.2* (19.5 ± 2.4)	1 ± 0.5	0 ± 0.5

^a^In parentheses, both airway and oesophageal pressures are shown in reference to tank pressure to enable calculation of transpulmonary pressures (airway pressure - oesophageal pressure) in reference to both atmospheric and body surface pressure inside the tank. Transrespiratory system pressures during CENPV (airway pressure - tank pressure) were compared to airway pressures during CPPV. CENPV, continuous external negative-pressure ventilation; CPPV, continuous positive-pressure ventilation. *P = 0.03 and ^†^*P *= 0.04 compared to the corresponding inspiratory or expiratory value during CPPV.

### Haemodynamics

**Figure 4:**
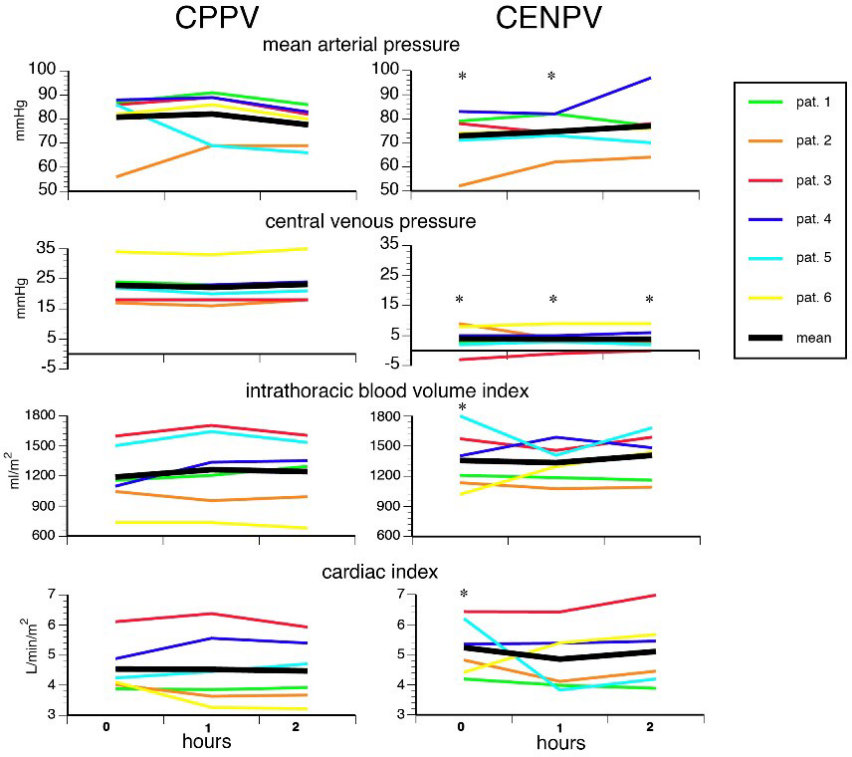
**Haemodynamics during continuous positive-pressure ventilation (CPPV) and continuous external negative-pressure ventilation (CENPV)**. Measurements were taken at time 0 (5 minutes after the recruitment manoeuvre) immediately after starting the 2-hour ventilatory period of CPPV or CENPV. The changes in intravascular pressure effects were more permanent in contrast to the more transient effects on intrathoracic blood volume and cardiac index. *P < 0.05 compared to corresponding values at 1 or 2 hours during CPPV.

Central venous pressures decreased more than arterial pressures during CENPV (Figure 4). Simultaneously, the intrathoracic blood volume index increased by 15% and the cardiac index increased by 20%, whereas no differences were found after 1 and 2 hours (Figure 4). After 2 hours of CENPV, the heart rate ranged between 57 and 126 beats/minute (median = 89 beats/minute), and the extravascular lung water index varied between 6 and 14 ml/kg (median = 9.5 ml/kg), and these parameters also did not differ between CPPV and CENPV. During the recruitment manoeuvre, no relevant impairments were observed and all changes returned to baseline immediately.

## Discussion

CENPV improved gas exchange considerably compared to CPPV, which was achieved at matched tidal and end-expiratory lung volumes with lower airway, intraabdominal and transpulmonary pressures, and at least initially improving haemodynamics. Values for inspiratory airway pressures during CPPV were similar compared with tank pressures during CENPV, although negative inspiratory tank pressures were reached only at end inspiration. The matching of end-expiratory lung volumes was obtained with an end-expiratory negative pressure of -15 cmH_2_O, which corresponded to a PEEP value of 16 cmH_2_O in five of six patients. This constancy was surprising and probably indicates very similar degrees of lung injury. Concordantly, the patients had very similar PaO_2_/FiO_2 _values at a PEEP of 16 cmH_2_O immediately before the measurement period. With less efficient cuirass or poncho wrap systems, higher pressure values were necessary to achieve the same end-expiratory lung volumes compared to positive pressures [11,12]. Concordantly, when these systems were used to apply continuous negative pressure during IPPV in patients with lung injury, gas exchange either did not improve [11,12] or even deteriorated when end-expiratory lung volumes were not matched [13]. We matched lung volumes, and the PaO_2_/FiO_2 _ratio increased impressively during CENPV, which may indicate alveolar recruitment with a decrease in pulmonary shunting.

On the contrary, even relatively high PEEP values used in the present study were apparently still insufficient to maintain recruited lung volumes during CPPV. In surfactant-depleted rabbits, Grasso *et al. *also matched end-expiratory lung volumes and observed better oxygenation during CENPV than during CPPV [7]. This effect was associated both with more aerated lung tissue and less lung injury after 2.5 hours with the use of high tidal volumes of 12 ml/kg [7]. In our pilot study, we measured neither aerated lung tissue nor FRC and we did not assess markers of lung injury. However, we speculate that, similarly to the observations of Grasso *et al. *[7], the improved oxygenation during CENPV observed in the present study could also be associated with alveolar recruitment and an increase in FRC. Increased FRC during CENPV would reduce alveolar strain (ratio between tidal volume inflated and FRC) [21], and improved lung recruitability during CENPV might be associated with less injurious intratidal alveolar opening and closing of lung tissue [22]. Grasso *et al. *assumed that CENPV might be more effective and less injurious because of more homogeneous distension of the lung as negative pressure is distributed across a broad surface of the chest wall and abdomen [7].

Concordantly, at given levels of transpulmonary pressure, Grasso *et al. *observed greater end-expiratory volumes, and transpulmonary pressures were lower when corresponding positive and negative pressure values were compared [7]. Our data seem to confirm their observations, although we did not measure transpulmonary pressures under static conditions. Therefore, and because we did not measure changes in transpulmonary pressure at 0 cmH_2_O at expiration as described by Chiumello *et al. *[21], we did not assess the global average lung stress in the present pilot study. Similarly to other experimental data [7], however, transpulmonary pressures were lower during CENPV at end inspiration; therefore, we speculate that lung stress could be lower as well in comparison to CPPV. Concordantly with the observations made by Grasso *et al. *[7], our data also suggest that the development of transpulmonary distending pressures may substantially differ during CENPV compared to CPPV. This may be associated with different regional pleural pressure gradients throughout the lungs that are poorly represented on the basis of just one value of transpulmonary pressure [7].

During CENPV, transrespiratory system pressures (TRP) (airway pressure minus tank pressure) were about 3 cmH_2_O lower at inspiration and about 3 cmH_2_O higher at expiration as endotracheal airway pressures became positive during CENPV, probably due to the expiratory resistance of the endotracheal tube. These slightly higher TRP values at expiration may be sufficient to explain the improved oxygenation observed during CENPV.

The TRP differences between CENPV and CPPV, which varied considerably at inspiration and expiration, might be cause by different distributions of positive and negative pressures, depending on individual differences in pulmonary mechanics. Despite our study design, in which we used matched tidal volumes and randomization of ventilatory modes, we found the same TRP value of 31 cmH_2_O in three patients, which may also reflect very similar degrees of lung injury.

The high intraabdominal pressures [19] decreased by 20 mmHg during CENPV. This may counteract the effects of high intraabdominal pressures such as cranial shifts of the diaphragm with consequent lung volume reduction, reduced lymphatic flow and lung oedema formation [23]. Intraabdominal perfusion pressure improved as the mean arterial pressure decreased only by 10 mmHg, which could be beneficial, especially when visceral blood flow is impaired.

In comparison to arterial pressures, the quite elevated central venous pressures decreased more extensively during CENPV, indicating the relatively greater impact on the venous circulation than on the arterial circulation, where the vessel tone is stronger. When central venous pressures decreased during CENPV, the high intrathoracic blood volume indices further increased by 15%, reflecting improved venous return, and the cardiac index improved considerably by 20%. Compared to CPPV, this improved venous return may result in less alteration of mixed venous oxygen content and therefore may contribute to maintaining higher levels of arterial oxygen content during CENPV.

In the present study, the heart rate also remained unchanged, indicating that the greater transpulmonary blood flow during CENPV apparently resulted from higher stroke volumes. Borrelli *et al. *observed very similar increases in cardiac output at lower intrathoracic blood volumes when a poncho was used to apply continuous negative pressure during IPPV [12]. The greater preload in the present study outweighed the effects on afterload, which increased when intrathoracic pressures decreased. During CENPV, both central venous and intraabdominal pressures decreased, which could result in similar pressure gradients for venous return compared to CPPV. In agreement with this finding, Grasso *et al. *did not observe changes in cardiac output when a whole-body device was used, but they did when negative pressure was applied to the chest only [7].

CENPV has been suspected to increase extravascular lung water compared to CPPV as a result of more negative pleural and interstitial pressures and because of higher left ventricular filling after enhanced venous return [24,25]. In our present study, extravascular lung water indices did not differ during CENPV compared to CPPV, which has also been observed in experimental studies [7,24,25]. Lung water can increase with PEEP by decreasing lung lymph flow, which has been attributed to compressed pulmonary lymphatic vessels [26]. These compressions do not occur under CENPV and may outweigh other effects, resulting in lung water values similar to those associated with CPPV.

The very small number of patients in this study represents its main limitation and primary source of errors. In four of the six patients, pulmonary aspiration of gastric content was either the main or one contributory predisposing factor in the development of ARDS. Extrapolating this observation to patients with other predisposing factors must be done with caution. In any case, the considerable variation in PaO_2_/FiO_2 _responses to CENPV was apparently independent of the underlying disease, the efficiency of the prior lung recruitment manoeuvre or the severity of lung injury. Interestingly, increased oxygenation in response to placement in the prone position also was not related to lung recruitability in response to positive pressures [27]. Finally, at this stage the reasons for the different individual responses to CENPV remain unclear.

As a possibly less injurious and more effective mode of ventilation, CENPV appears especially attractive when the potential to eliminate endotracheal intubation is taken into consideration. Tank respirators were used decades ago to apply continuous external negative pressure in three patients with severe pneumonia who were not intubated [8-10]. This improved oxygenation and enabled maintenance of spontaneous breathing in severe lung injury.

## Conclusions

Our results demonstrate for the first time that CENPV is applicable and effective, even in severely critically ill patients in a modern intensive care setting. The present study confirms recent experimental data and encourages consideration of further studies of the physiological effects and clinical effectiveness of CENPV in patients with ARDS.

## Key messages

• CENPV differs substantially from CPPV and improves oxygenation under more physiologic conditions in patients with ARDS.

## Abbreviations

ARDS: acute respiratory distress syndrome; CENPV: continuous external negative-pressure ventilation; CPPV: continuous positive-pressure ventilation; FiO_2_: fraction of inspired oxygen; FRC: functional residual capacity; ECG: electrocardiogram; IPPV: intermittent positive-pressure ventilation; paO_2_: arterial oxygen tension; paCO_2_: carbon dioxide tension; PEEP: positive end-expiratory pressure; PiCCO: Pulse Contour Cardiac Output; SAPS: Simplified Acute Physiology Score; TRP: trans-respiratory system pressure.

## Competing interests

The authors declare that they have no competing interests.

## Authors' contributions

KR, UM, MC, WK and AC participated in the design of the study; KR, UM and MC participated in the collection and the assembly of the data; KR and TD performed the statistical analysis; BS, JA, CW and AC gave technical or logistical support; KR, UM, TD, JA, CW and AC helped to draft the manuscript and, all authors read and approved the final manuscript.
